# Investigation into the Mechanical Properties of Swimming-Goggle Gaskets After Prolonged Water Conditioning

**DOI:** 10.3390/ma19163508

**Published:** 2026-08-19

**Authors:** Paulina Maślanka, Laura Kozanecka, Ryszard Korycki, Halina Szafrańska

**Affiliations:** 1 Department of Mechanical Engineering, Informatics and Chemistry of Polymer Materials, Lodz University of Technology, 90-924 Lodz, Poland; ryszard.korycki@p.lodz.pl; 2 Lodz University of Technology, 90-924 Lodz, Poland; 245313@edu.p.lodz.pl; 3 Department of Chemistry, Casimir Pulaski Radom University, Stasieckiego 54, 26-600 Radom, Poland

**Keywords:** swimming-goggle conditioning, gasket material properties, numerical simulation and tests

## Abstract

This study combines experimental conditioning and numerical simulations to evaluate water-induced degradation of swimming-goggle gaskets and their mechanical response under representative loading conditions. Two gasket geometries were modeled, with the flatter profile serving as a reference and the more anatomically representative variant analyzed using finite-element analysis for neoprene, silicone, and SEBS materials of different Shore A hardness under prescribed periorbital pressure distributions. Commercial gaskets were conditioned by static immersion in chlorinated, distilled, and saline water for 20, 30, and 40 days, followed by measurements of Shore A hardness, breaking force, and elongation at break. The numerical results showed that gasket geometry substantially influences the predicted deformation patterns relevant to facial conformability, despite similar overall deformation magnitudes. Prolonged water exposure caused considerable material degradation. The breaking force decreases by approximately 48% after 20 days and by about 50% after 40 days of conditioning, irrespective of the environment. Elongation at break changed by approximately 3–4% in chlorinated and distilled water, whereas saline water caused reductions of approximately 11% after 20 days and 36% after 40 days. Additionally, the hardness of the material drops by about 10–13% after 20 days and subsequently remains almost constant. In the adopted cyclic loading–recovery simulation, silicone exhibited substantially lower residual deformation than SEBS. Overall, the results demonstrate the importance of material selection and geometry optimization and provide a comparative basis for further studies combining environmental conditioning with experimentally calibrated numerical models.

## 1. Introduction

**Figure 1:**
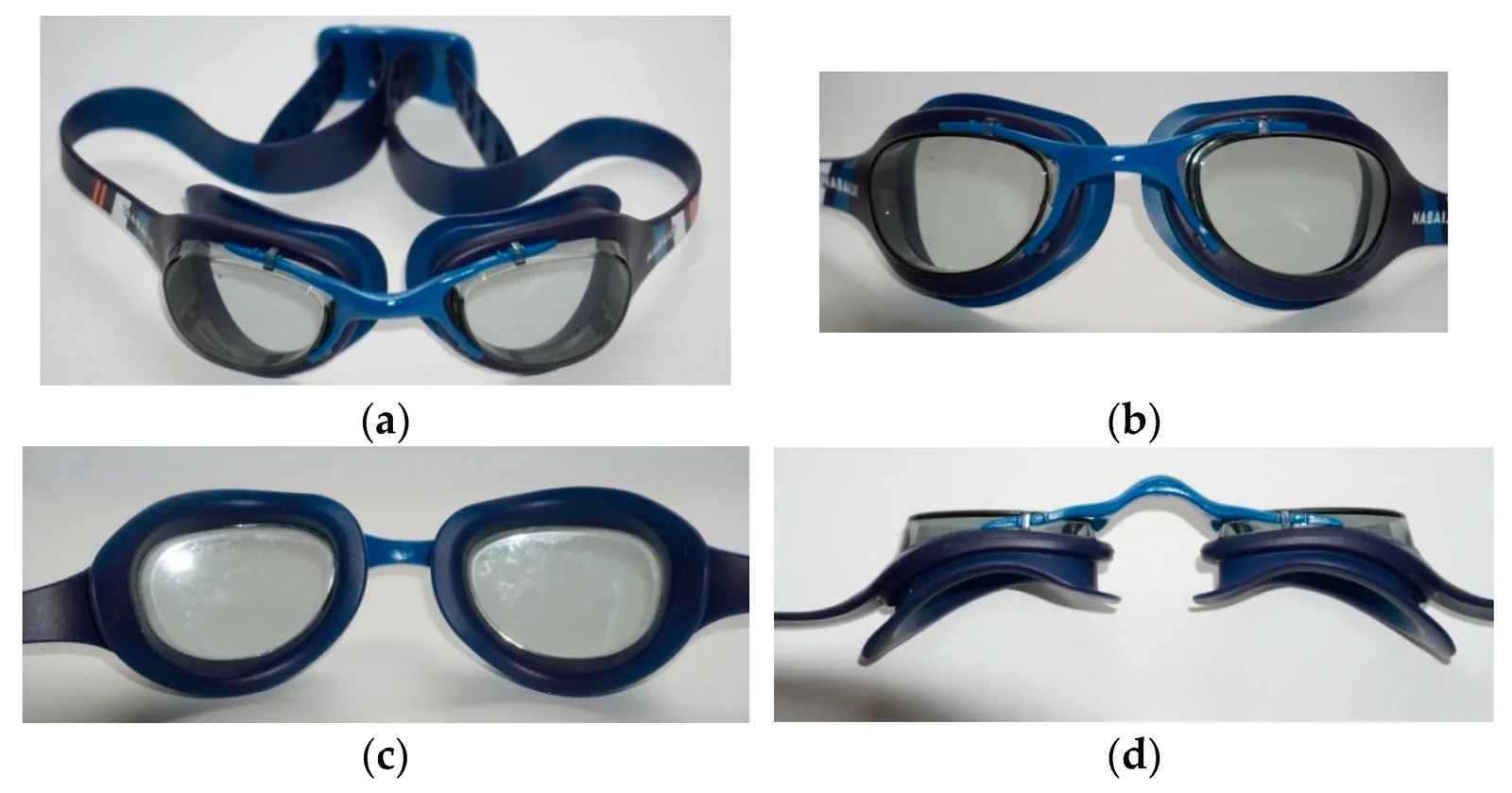
Nabaiji Xbase swimming goggles: (**a**) general view; (**b**) front view; (**c**) rear view; (**d**) top view.

Goggles are necessary swimming accessories for training or swimming in a pool. Soft goggles are designed for recreational swimming. They are widely available and inexpensive and have a simple design. Hard goggles have a rigid and often modular structure that allows for a custom fit to the face shape. They provide greater comfort and a better seal; however, they are expensive and used mainly by professionals. Semi-hard goggles offer a compromise between these two types. Swimming for a long time with leaky goggles is uncomfortable because the eyes are constantly exposed to disinfectants in the water. Therefore, long-lasting comfort during use is crucial for the user. Soft Nabaiji Xbase swimming goggles (Figure 1), an entry-level model, were selected for the analysis; they are widely used, readily available, and relatively susceptible to aging.

Xinkai Ding et al. [1] examined the synergistic effects of surface texture and material aging on the sealing reliability of marine seals. Material aging significantly reduced contact pressure and von Mises stress, leading to a deterioration in sealing reliability. Conversely, surface textures enhanced contact pressure distribution and mitigated performance degradation. Wang et al. [2] proposed an aging life evaluation method for sealing rings based on a sealing performance degradation model and an artificial neural network model. Sealing rings are the core components of flange sealing structures and play a crucial role in the storage and operation of gas generators (Zhu et al., [3]). In this context, the effect of rubber aging on sealing performance was investigated using nonlinear finite-element analysis. The suitable aging temperature ranges for different materials of mechanical seal rings are explored by Li et al. [4]. The experimental results reveal a clear trend: as the aging temperature rises, the degree of end-face deformation of the sealing ring gradually intensifies, reducing the smoothness of the sealing interface. The experimental results confirm that the reciprocating temperature cycle causes a type of fatigue failure, which could result in an increase in the rubber compression set (Sen Li et al., [5]). The accelerated aging test method was proposed based on the dominant damage mechanism of the rubber material caused by the temperature cycle treatments.

The influence of oil and thermal aging on the characteristics of nitrile butadiene rubber seals was studied by coupling the mechanical with the tribological behavioral changes of NBR in oil and the thermal environment (Li et al., [6]). It can be seen from the results that the elastic modulus increased with aging time in the thermal oxidative aging testing.

Most of the swimming goggles are designed as one-size-fits-all, which may cause improper fit to a wearer’s facial shape. The detailed design characteristics of lens, strap, gasket, and nose bridge of 26 commercialized swimming goggles were reviewed and the dimensions of five swimming goggles summarized by Park et al. [7]. Next, 3D scanning was employed to customize the goggles in a CAD environment.

Swimming goggles still face numerous challenges in practical use, including deterioration and failure of anti-fog coatings, residual water marks on lens surfaces, and relatively short service life in complex environments. To enhance the performance of swimming goggles, Ding et al. [8] employed a combination of plasma cleaning and mechanical spraying methods, thereby fabricating lenses with superhydrophobic properties. Lenses exhibit different refractive power issues depending on the medium. The change in refractive power between air and water can be addressed by replacing a plano-concave lens with a plane Fresnel lens of a specific diopter (Yeh et al., [9]).

Swimming-goggle gaskets can be made of neoprene, silicone, or SEBS. The fundamental laboratory test in rubber research is the uniaxial tensile test. This test is performed in accordance with the current EN-ISO 37 standard [10], which strictly specifies the testing conditions and the dimensions of the specimens [11].

The environmental impact of swimming goggles can be evaluated through a life cycle assessment (LCA), comparing virgin and recycled polycarbonate models (Nikonova et al., [12]). The LCA was modeled using SimaPro 9.6.0.1 with the Environmental Footprint (EF) 3.1 method to analyze 16 impact categories.

Intraocular pressure, defined as the fluid pressure inside the eye, serves as an indirect but well-established proxy for the compressive force exerted by the gasket on the periorbital tissues. However, it is important to note that the actual contact pressure at the skin–gasket interface is significantly greater than the resulting IOP elevation. The difference arises from the mechanical damping and stress distribution through the soft facial tissues (skin, subcutaneous fat, and orbital contents), which absorb and redistribute part of the applied force before it is transmitted to the globe. Typical IOP elevations range from 2 to 5 mmHg (≈267–667 Pa) for comfortable and secure fits, with higher values (up to 10 mmHg or more) observed in tighter or smaller goggles [13,14,15].

The aim of this paper is to combine experimental conditioning and numerical simulations to evaluate water-induced degradation of swimming-goggle gaskets and their mechanical response under representative loading conditions. Numerical simulations were performed to determine the material behavior and deformation of the gaskets, using parameters corresponding to degraded material. The experimental measures used to assess material impact included hardness, tensile strength, and relative elongation at break determined by means of an Instron testing machine. The results will help evaluate the resistance of the materials under simulated operating conditions and provide recommendations for enhancing their properties. This may be particularly significant for sports equipment manufacturers when selecting gasket materials. To the best of our knowledge, the quantification and numerical modeling of water-induced swimming-goggle gasket degradation have not been documented in the literature. Therefore, these elements can be considered the novel contributions of the article.

## 2. Materials

Swimming goggles, as a product intensively used in aquatic environments, must meet a wide range of requirements. They should be resistant to pool, sea, and distilled water, while ensuring flexibility and long-term durability. A key component in achieving these objectives is the gasket, which is designed to ensure watertightness and long-lasting comfort. The gaskets are most commonly made of compliant and elastic materials.

A typical material used is SEBS (styrene–ethylene–butylene–styrene), a thermoplastic elastomer belonging to the block copolymer family, in which rigid styrene segments are chemically bonded with flexible ethylene–butylene copolymer segments. Through hydrogenation, the material gains high thermal stability and chemical resistance, particularly to aquatic environments, ozone, and UV degradation [16]. The structure consists of alternating styrene blocks, which provide hardness and mechanical strength, and flexible ethylene–butylene blocks that impart rubber-like elasticity. SEBS offers a very wide range of hardness levels (Table 1), allowing for a precise fit to facial anatomy and ensuring watertightness. Typical tensile strength can be further enhanced with PP additives, while excellent compression-set resistance ensures that the goggles’ seal remains effective over time [17,18].

Neoprene is a synthetic rubber (polychloroprene) characterized by high resistance to water, mechanical damage, and temperature fluctuations. It serves as an effective thermal insulator and is completely waterproof. The material maintains its elasticity, abrasion resistance, and elongation across a wide temperature range (Table 1) [19]. Neoprene exhibits high resistance to oils, organic solvents, and weather conditions, including UV radiation. It is a key material in water sports, particularly for goggle seals [20]. Its closed-cell structure prevents air flow and does not wick away sweat, which may lead to dermatological issues. Thin neoprene (under 2 mm) is relatively flexible; however, thicker variants become rigid, especially in cold environments. Neoprene resists degradation better than conventional rubber, making it well suited for demanding applications such as goggle gaskets.

Silicones are organosilicon polymers that can take the form of an elastomer. Silicone is soft, adapts well to the anatomical shape of the face, and does not absorb water, ensuring a tight seal. Pressure on the skin around the eyes is minimal. The material is resistant to chlorine, sea salt, and UV radiation; it does not become brittle and is resistant to aging. Silicone is physiologically inert and rarely causes allergic reactions. The material possesses good mechanical properties (Table 1) [16,21]. Currently, silicone is the most popular material used in swimming-goggle seals due to its optimal balance between comfort and durability.

## 3. Methods

### 3.1. Numerical Analysis

The numerical model was developed and solved in ANSYS 2025 R1 Mechanical as a geometrically nonlinear static analysis of deformations and stresses in the swimming-goggle gasket under prescribed surface loading that represents strap tension. Facial geometry, compliant facial tissues and explicit gasket–face contact were omitted, and hydrostatic fluid pressure was not considered.

#### 3.1.1. Geometry

**Figure 2:**
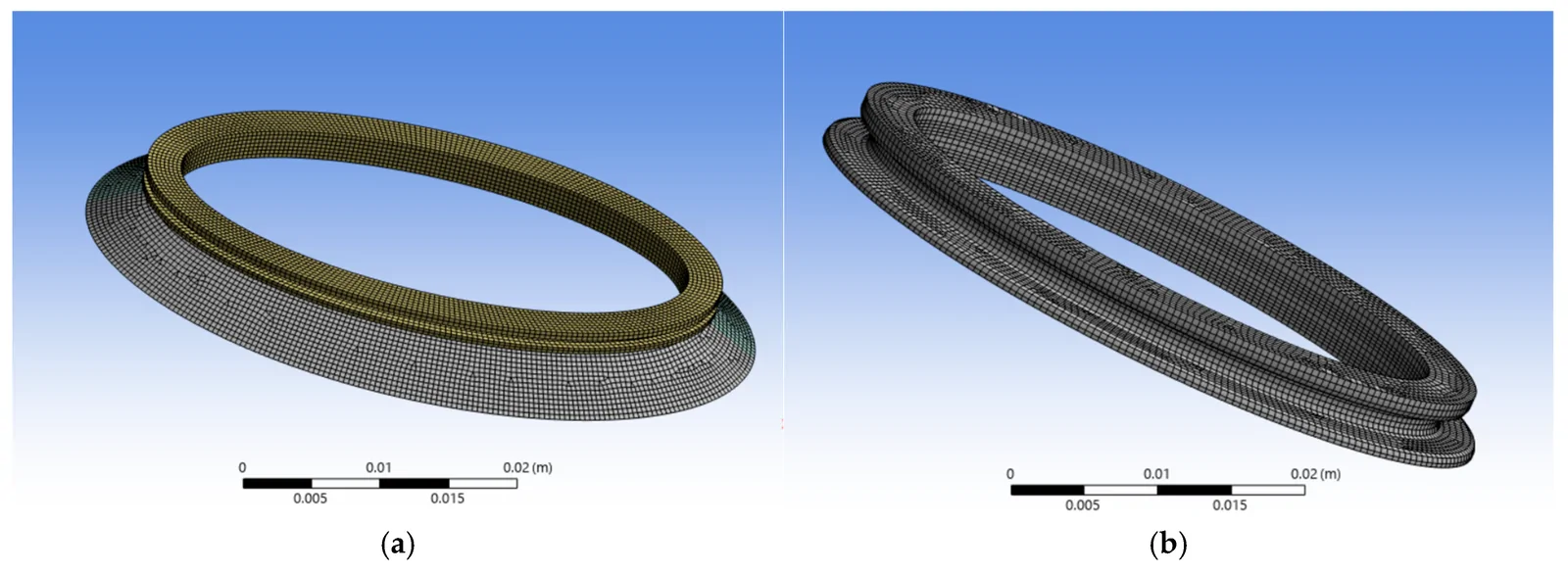
Visual representation of swimming-goggle seals’ geometric variants: (**a**) Variant 1; (**b**) Variant 2.

Two geometric types of swimming-goggle seals were modeled and imported from DesignModeler in .agdb format, as described in Table 2 and Figure 2. Total volume values representing both geometries are in the range of general sizes of gaskets for swimming goggles.

Variant 2 represents a flatter gasket profile, less conforming to the orbital anatomy compared to Variant 1. Therefore, it is treated as a reference (baseline/signal) configuration, for which calculations were performed using only SEBS and a uniform pressure distribution.

Variant 1, considered more representative of real-world gasket geometry, was analyzed across a broader range of cases, including three different materials (silicone, SEBS 65, SEBS 50) and different pressure distributions further described in Section 3.1.3.

#### 3.1.2. Finite-Element Mesh

Meshing was performed using the MultiZone method, with preference for hexahedral elements in mappable regions (Mapped/Swept Type = Hexa).

For Geometric Variant 1, the global element size was approximately 1.50 mm, while a local body sizing control of 0.40 mm was applied to the gasket region. The final mesh comprised 19,439 nodes and 14,165 elements. Mesh quality was evaluated using the skewness metric with an average value of 0.176, whereas minimum and maximum values equaled 5.631 × 10^−4^ and 0.458, respectively, with standard deviation 0.124.

For Geometry Variant 2, the global body sizing with adaptive refinement was applied along with a local body sizing control of 0.50 mm. The final mesh comprised 25,148 nodes and 19,319 elements. The resultant skewness metric had an average value of 0.235, whereas the minimum and maximum values were 5.062 × 10^−3^ and 0.812, respectively, with standard deviation 0.207.

Both meshes satisfy quality criteria for large-deformation hyperelastic analysis, although Variant 2 exhibits a higher maximum skewness value. The maximum skewness value below 0.5 (Geometry Variant 1) indicates high mesh quality.

#### 3.1.3. Boundary Conditions and Loading

Three gasket materials were investigated in this study: neoprene rubber Shore A 40, silicone Shore A 40, and SEBS (styrene–ethylene–butylene–styrene) with Shore A hardness ranging from 20 to 65. Shore A hardness values from 20 to 65 were considered to examine a broad range of material stiffnesses. The Shore A 20 and Shore A 65 variants represented the lower and upper bounds of the analyzed stiffness range, respectively.

All material parameters were adopted from the literature [22,23,24]. Hyperelastic behavior was described using the Neo-Hookean model, with parameters listed in Table 3.

The parameters listed in Table 3 should be regarded as nominal material representations rather than as material-specific calibrations of the tested commercial gaskets. Elastomer stiffness and nonlinear response may vary with formulation, processing history, aging, and manufacturer. The parameters for silicone, neoprene, and the remaining material cases were adopted from the literature to provide a consistent basis for comparative analysis.

The Neo-Hookean model was selected as a simple first-order hyperelastic formulation for the comparative analysis of the considered gasket materials and geometries. The use of a common one-parameter formulation enabled the relative influence of material stiffness and gasket geometry to be evaluated consistently, despite the limited availability of material-specific experimental data. It is generally suitable for representing the initial and moderate-strain response of rubber-like materials, although its quantitative accuracy depends on the specific material and loading mode.

The material parameters for silicone, neoprene, and the remaining material cases were adopted from the literature. For the reference SEBS material, the initial stiffness was additionally estimated from the available graphical uniaxial tensile-to-failure response and the corresponding specimen information. Because the original numerical test data were unavailable, the available curve could be used only for an approximate estimation of the initial stiffness and not for a formal fit of the complete nonlinear response.

In the present study, the maximum equivalent strain under the lower nominal pressure condition of 5 kPa was approximately 5.3%, while higher values occurred only locally under severe or transient loading conditions. However, localized peak values may be more sensitive to the selected constitutive formulation and should therefore be interpreted with caution. The model is used primarily to compare the relative responses of the materials and geometries rather than to provide a material-specific prediction of extreme local strain.

**Figure 3:**
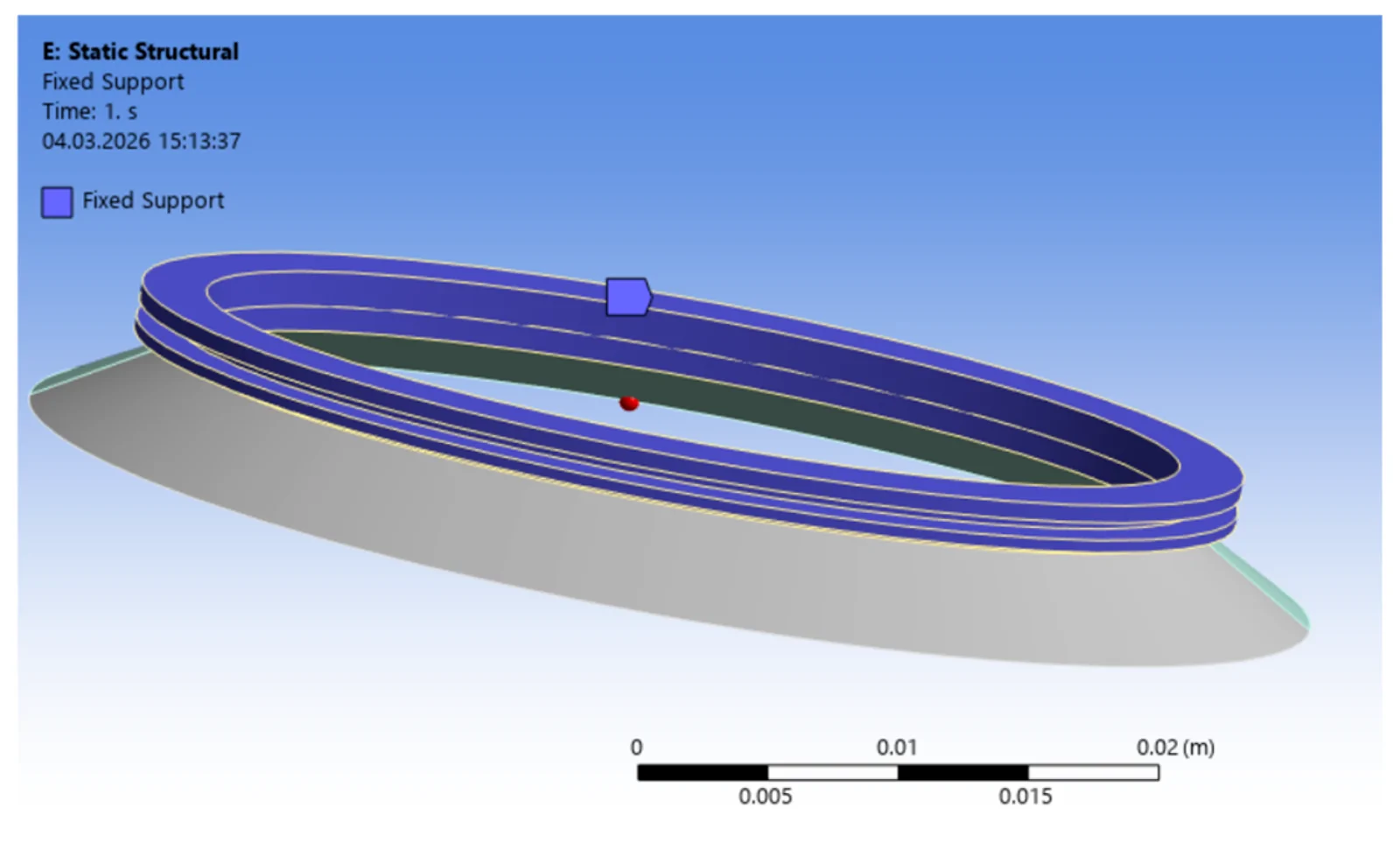
Fixed Support application.

The analyses were performed in the Static Structural module with large deflection effects enabled (Large Deflection = On). Boundary conditions consisted of Fixed Support applied to selected faces, representing rigid attachment to the goggle frame and strap connection points (Figure 3).

The applied loading represented the resultant mechanical action associated with strap tension and interaction with the periorbital region. Facial geometry and compliant facial tissues were not explicitly modeled; instead, prescribed surface pressure was applied to the inner gasket surfaces. This idealization is consistent with the design principle of swimming-goggle gaskets, which are intended to distribute contact pressure over the periorbital region [25]. The applied values should therefore be interpreted as nominal equivalent pressures rather than contact pressures predicted by the model. This simplification does not account for soft-tissue deformation, friction, progressive establishment or loss of contact, or pressure redistribution caused by individual facial curvature. Accordingly, the model was used primarily to compare gasket materials and geometries under identical assumed loading conditions and does not directly predict local skin pressure, actual contact area, or sealing performance. The constant-pressure cases represent idealized post-donning conditions, whereas the variable-pressure case represents a simplified non-uniform loading scenario rather than a complete simulation of the donning process.

The contact pressure acting on the gasket–skin interface was estimated from the tension generated in the goggle strap using a simplified linear elastic approach based on the effective Young’s modulus of the strap material (with SEBS Shore A 40 as reference). The goggle strap was stretched to different lengths corresponding to comfortable and tight fits. The elongation Δ*L* was measured relative to the unstretched reference length *L*_0_ of the strap segment between the attachment points on the goggle frame. Similar approaches were described in [26,27].

The tensile stress *σ* in the strap was calculated using Hooke’s law (1) and (2).

(1) σ=E·ε;

(2) $$\varepsilon =\frac{∆L}{L_{0}},$$

where σ is stress [Pa], *E* is the effective Young’s modulus of SEBS Shore A 40 [Pa], ε is engineering strain [-], ∆L is the difference between stretched length and reference length [m], and $L_{0}$ is the reference length [m].

The tensile force in the strap was then obtained by multiplying the stress by its cross-sectional area (3). The average contact pressure on one gasket–skin interface was calculated as in (4).

(3) $$F=\sigma \cdot A_{strap};$$

(4) $$P=\frac{F}{A_{contact}},$$

where F is total force [N], $A_{strap}$ is the cross-sectional area of the swimming-goggle strap [m^2^], P is contact pressure on one gasket–skin interface [Pa], and $A_{contact}$ is the gasket–face contact area [m^2^].

The measured strap lengths, calculated strains, forces, and resulting contact pressures are summarized in Table 4. The value of 5 kPa corresponds to a comfortable and secure seal under typical real-world strap tension, while 25 kPa represents a tight fit associated with excessive compression and discomfort. These pressures, estimated using measured strap dimensions and elongations together with assumed material stiffness and nominal loading area and supported by literature data [28], were applied as nominal equivalent loads in the simulations (Table 4).

**Figure 4:**
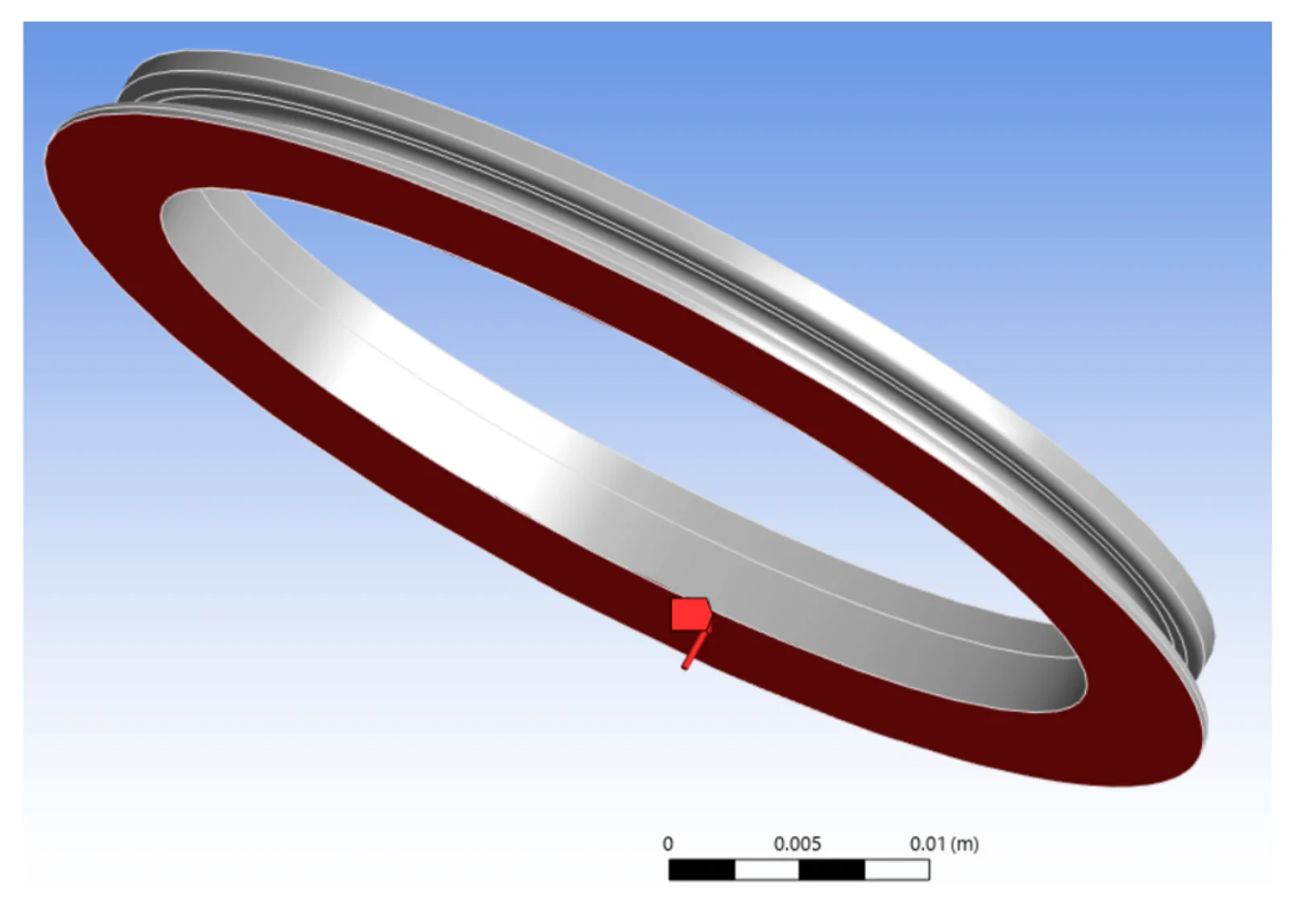
Constant pressure application.

In the first case, a constant distributed pressure was applied to the inner gasket surfaces (Figure 4).

**Figure 5:**
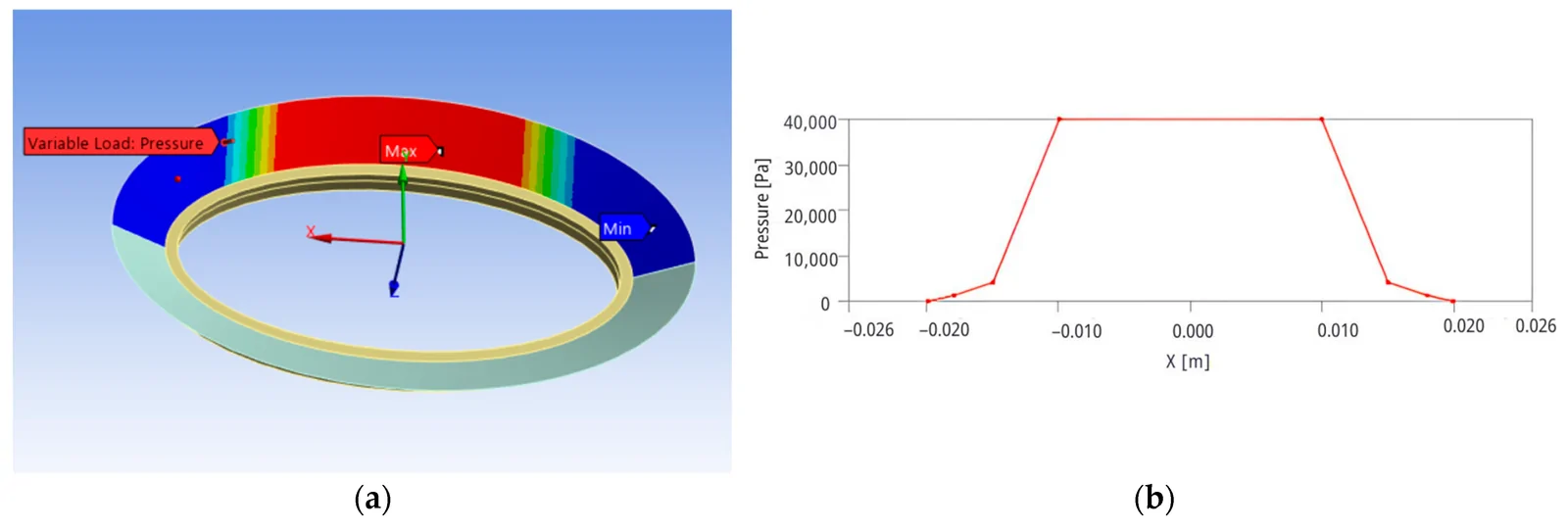
Variable pressure: (**a**) application; (**b**) distribution along *x*-axis.

Another case (Figure 5) applies an idealized non-uniform pressure distribution intended to represent a possible top-down loading sequence during gasket placement.

Simulations with constant and variable pressure were performed for neoprene rubber Shore A 40, silicone Shore A 40, and SEBS Shore A 40. For the full range of SEBS materials (Shore A 20–65), only the two constant pressure levels were considered.

To evaluate the influence of uncertainty in the assumed SEBS stiffness on the predicted structural response, an additional material sensitivity analysis was performed under a uniform pressure of 25 kPa, representing the more severe of the two uniform-pressure conditions. Three SEBS stiffness variants were considered: a soft Shore A20 variant, the experimentally informed reference variant, and a stiffer Shore A40 variant. The reference material parameters were estimated from the available experimental uniaxial tensile-to-failure curve and the corresponding specimen data. Because the original numerical test data were unavailable, the initial portion of the force–elongation curve was digitized from the supplied plot and used to estimate the initial material stiffness, resulting in an initial shear modulus of approximately *μ* = 0.40 MPa, corresponding to *C*_10_ = 0.20 MPa. All variants were represented using a nearly incompressible Neo-Hookean model, while the geometry, mesh, boundary conditions, contact definitions, and loading conditions were kept unchanged. The sensitivity assessment included the maximum reaction force *F*_max_, displacement at a common reference force *u*(*F*_ref_), volume-weighted mean compressive strain ${\bar{c}}_{V}$, and volume fraction exceeding 30% compression *f*_30_.

The experimental conditioning results were not directly introduced into the finite-element model as aging-dependent constitutive parameters. The hardness, breaking force, and elongation-at-break measurements were used to quantify the degradation trends of the commercial SEBS gaskets. However, the available experimental data were insufficient to identify complete hyperelastic material parameters separately for each conditioning medium and exposure period. The experimentally informed reference SEBS stiffness was therefore used only in the material sensitivity analysis and should not be interpreted as a constitutive representation of the material after conditioning.

The solution was obtained in a single time step with linearly ramped loading up to the final value at *t* = 1 s. The Mechanical APDL solver was used with force and displacement convergence monitoring.

Additionally, a cyclic relaxation analysis was performed for SEBS Shore A 40 and silicone Shore A 40. This simulation consisted of ten time steps representing five cycles of loading and recovery: 1 h under a constant pressure of 25,000 Pa followed by 23 h of relaxation (zero pressure). In this case, a linear viscoelastic material model was used, defined by Young’s modulus, Poisson’s ratio, and the Prony shear relaxation series.

The silicone and SEBS simulations were performed using the same gasket geometry, mesh, boundary conditions, pressure level, and loading–recovery history. The materials differed only in their linear viscoelastic parameter sets. The adopted silicone model had a total relative shear-relaxation amplitude of Σ*g_i_* = 0.33, corresponding to a long-term modulus ratio of (*G*_∞_/*G*_0_ = 0.67), and a maximum relaxation time of 10,000 s. For SEBS, the corresponding values were (Σ*g_i_* = 0.70), (*G*_∞_/*G*_0_ = 0.30), and 144,000 s. Volumetric relaxation was neglected. These nominal parameter sets were used for comparative purposes and were not calibrated specifically for the commercial gasket materials examined experimentally.

### 3.2. Conditioning

Synergistic effects of water and UV radiation cause structural changes in the material, reducing its elasticity, strength, and resistance to cracking. The commercial gaskets investigated in this study were made of styrene–ethylene–butylene–styrene (SEBS).

The degradation of these properties affects not only user comfort but also the safety and durability of the goggles. Training data from the Academic Sport Association indicate that the gaskets are the most vulnerable part when exposed to water. Material destruction along the side edges typically manifests after 1 to 1.5 years of operation. Material fatigue from repeated use and variable swimming loads causes gradual crumbling. Continuous loading of the gaskets throughout the swim accelerates their cumulative degradation.

To simulate the impact of different types of water, individual batches of glasses were conditioned by continuous immersion under normal conditions, i.e., at room temperature and with access to daylight. The conditioning temperature was maintained at 25 °C, corresponding to the minimum water temperature specified for an indoor sports swimming pool in the current World Aquatics Competition Regulations. The samples were placed in direct natural daylight adjacent to a window. Due to technical limitations, including the large number of samples and the need for their continuous rotation to ensure uniform irradiation, it was not feasible to establish a test setup using a xenon arc lamp for accelerated actinic aging. The conditioning was conducted in an enclosed room, and the environmental conditions were monitored periodically several times a day. The adopted parameters are as follows.

Three conditioning periods of 20, 30, and 40 days were selected to identify time-dependent trends in the material response. The protocol was designed to isolate the effects of prolonged exposure to different aqueous media. The conditioning periods represent laboratory exposure times and should not be interpreted as equivalents of specific periods of actual use. Actual service aging may additionally involve cyclic deformation, drying periods, sweat and sebum exposure, temperature variations, and controlled or variable UV radiation. For each aqueous medium and conditioning period, the samples were placed in hermetically sealed glass containers, each designed to hold four samples. The conditioning solutions were not replenished or replaced throughout the entire conditioning process.

Three different types of water were used, considering the potential use of the goggles in various water bodies: (1) swimming pool water (chlorinated water with chlorine concentration at the level of 0.8 mg/L, bound chlorine concentration 0.25 mg/L, and pH 7.0); (2) distilled water; and (3) saline water with a very high salinity of 35‰ (35 g of NaCl per 1 L of water), which corresponds to the salinity of the Mediterranean Sea. For comparison, the salinity of the Baltic Sea is approximately 7‰.

The tests were performed immediately after removal from the conditioning environment (approximately 1 min). The time elapsed between removal from the water and the commencement of the test had no impact on the material properties. The present conditioning procedure focused on static immersion aging and did not include continuous mechanical loading of the gaskets. Design specifications are currently being developed for a dedicated test stand intended to apply continuous dynamic loading to gasket materials throughout the entire aging process.

### 3.3. Testing Methods for Gasket Material Parameters

All experimental groups consisted of independent samples, with 20 measurements performed for each condition including the baseline state (without conditioning).

The hardness of the gasket material was measured using a standard Shore A analog durometer, model HBA 100-0 (0–100/1 ShA). Samples of the appropriate dimensions were cut immediately before the measurement. The first samples tested were those cut from the goggles without conditioning, the so-called zero samples.

To determine the optimal measurement location, the initial hardness was measured at several different points along the perimeter of the gasket. The samples have small dimensions, varying surface thicknesses, and small radii of curvature. The hardness was determined as the average of the measurements from different locations.

Material samples were tested for tensile strength using an Instron testing machine. Tensile loading was applied until the sample ruptured. The parameters selected to define the gasket strength were the maximum breaking force [N] and the relative elongation at break [%].

## 4. Results

### 4.1. Simulation Results

Nonlinear solution convergence was successfully achieved in all simulations according to force and displacement criteria, using the program-controlled Newton–Raphson method.

Results of deformation, stress, and strain under different loads of two geometry variants (Neoprene rubber, silicone, and SEBS Shore A 40—Variant 1; SEBS Shore A 40—Variant 2) are listed in Table 5 below.

**Figure 6:**
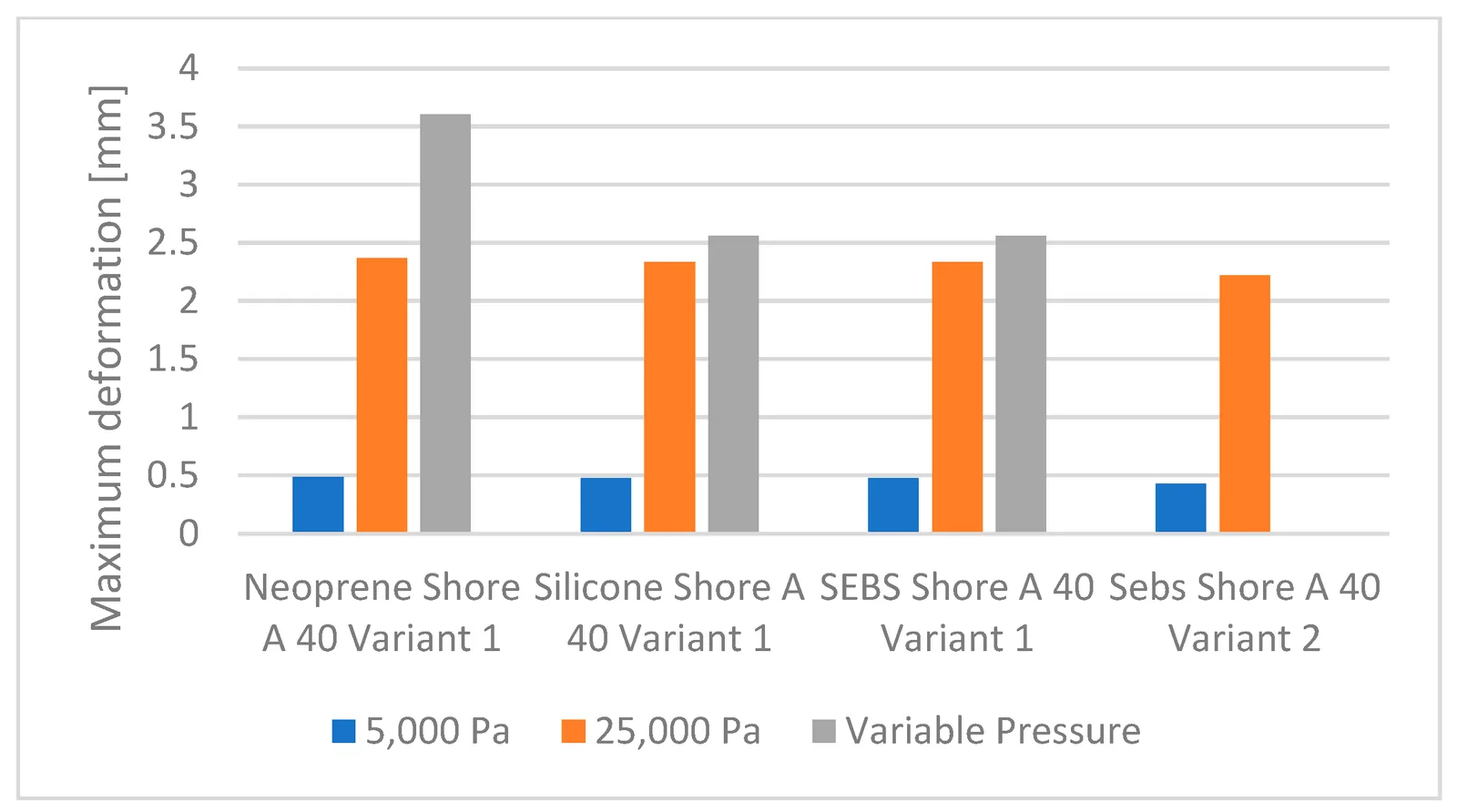
Maximum deformation of materials under different loads.

The gaskets exhibited highly consistent deformation behavior under constant pressure, with maximum displacements of approximately 0.5 mm at 5000 Pa and 2.3 mm–2.4 mm at 25,000 Pa across neoprene, silicone, and SEBS Shore A 40. Silicone and SEBS Shore A 40 displayed identical responses under identical loading. The largest deformation (3.6 mm) occurred for neoprene under the variable pressure distribution simulating the top-down donning process, as shown in Figure 6.

**Figure 7:**
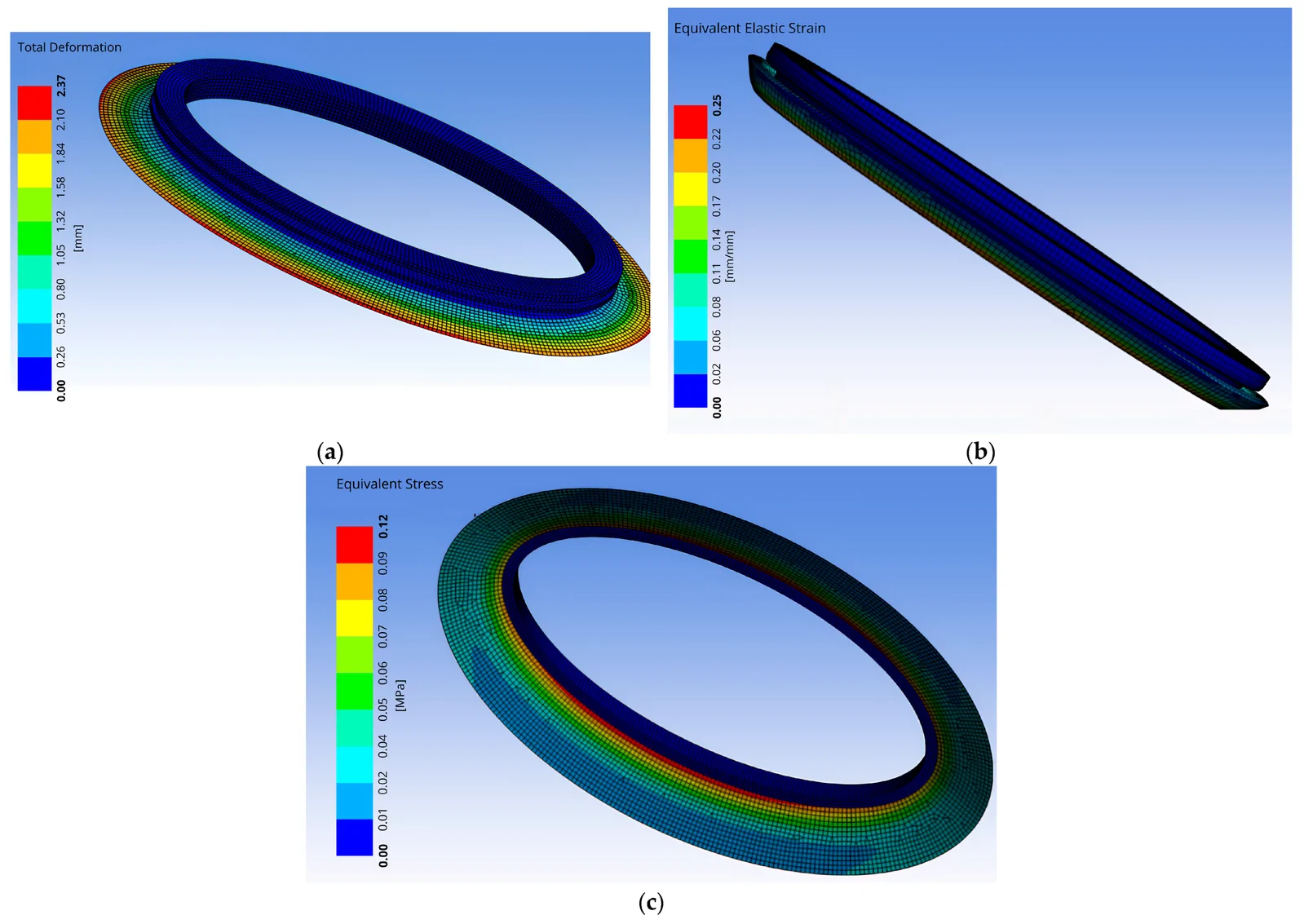
Constant loading visual results: (**a**) Variant 1 total deformation—neoprene Shore A 40 under constant pressure of 25,000 Pa; (**b**) Variant 2 equivalent elastic strain—SEBS Shore A 40 under constant pressure of 25,000 Pa; (**c**) equivalent stress—silicone Shore A 40 under constant pressure of 5000 Pa.

In all analyzed cases of constant loading, peak deformations were localized along the loaded inner edges of the gasket (Figure 7a); the simplified flat-face geometry in the model suggests that real facial curvature, particularly around the nasal bridge, would further intensify these local effects.

Although Variant 2 exhibited a slightly smaller maximum displacement, the deformation magnitude alone should not be regarded as an indicator of facial conformity. The observed difference should instead be interpreted in relation to the initial gasket geometry. Variant 1 has a pre-shaped three-dimensional skirt intended to accommodate the curvature of the periorbital region and retains a spatially contoured configuration under loading. In contrast, the flatter initial profile of Variant 2 results in a deformed configuration that appears less compatible with a curved anatomical surface. This qualitative comparison suggests that Variant 1 may have a greater potential to establish a continuous and more uniform gasket–face interface, whereas Variant 2 may be more susceptible to non-uniform conformity or local gaps. However, because explicit gasket–face contact and compliant facial tissues were not included, the present analysis does not directly quantify contact area, contact-pressure uniformity, leakage, or sealing performance.

Maximum von Mises stresses reached approximately 0.88 MPa for both silicone and SEBS, in all analyzed cases remaining concentrated in the central perimeter zone (Figure 7c) due to combined through-thickness compression and lateral expansion of the hyperelastic material.

Under the pressure corresponding to a comfortable and secure fit (5 kPa), the maximum equivalent strains remained at approximately 5.3%, while the average equivalent strains were below 1%. For the tight-fit condition of 25 kPa, representing excessive gasket compression and user discomfort, localized maximum equivalent strains increased to approximately 25–31%, whereas the average equivalent strains remained below 4%. The highest values, approximately 40–41%, occurred only as localized peaks under the non-uniform pressure distribution representing a transient top-down donning scenario. Thus, the high maximum strain values were confined to limited regions and to severe or transient loading conditions, whereas the overall gasket response was characterized by substantially lower average strains. The Neo-Hookean model was therefore considered a reasonable first-order approximation for evaluating the comparative response of the materials and geometries under normal operating conditions. Nevertheless, the localized peak values obtained under the tight-fit and variable-pressure scenarios should be interpreted with caution, as they extend beyond the moderate strain range in which the model provides its most reliable representation.

**Figure 8:**
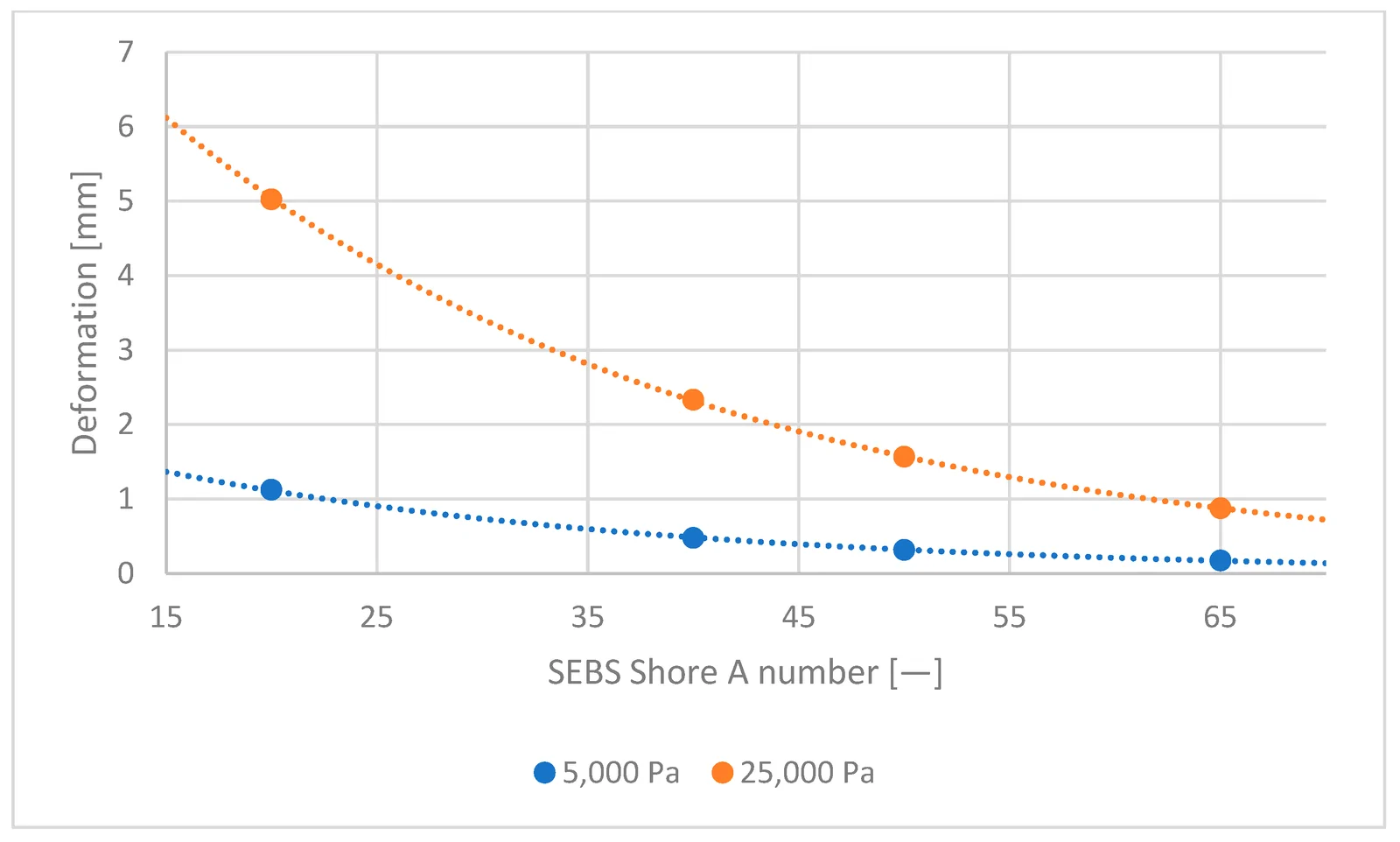
Total deformation of SEBS gaskets in the function of material stiffness (Variant 1).

**Figure 9:**
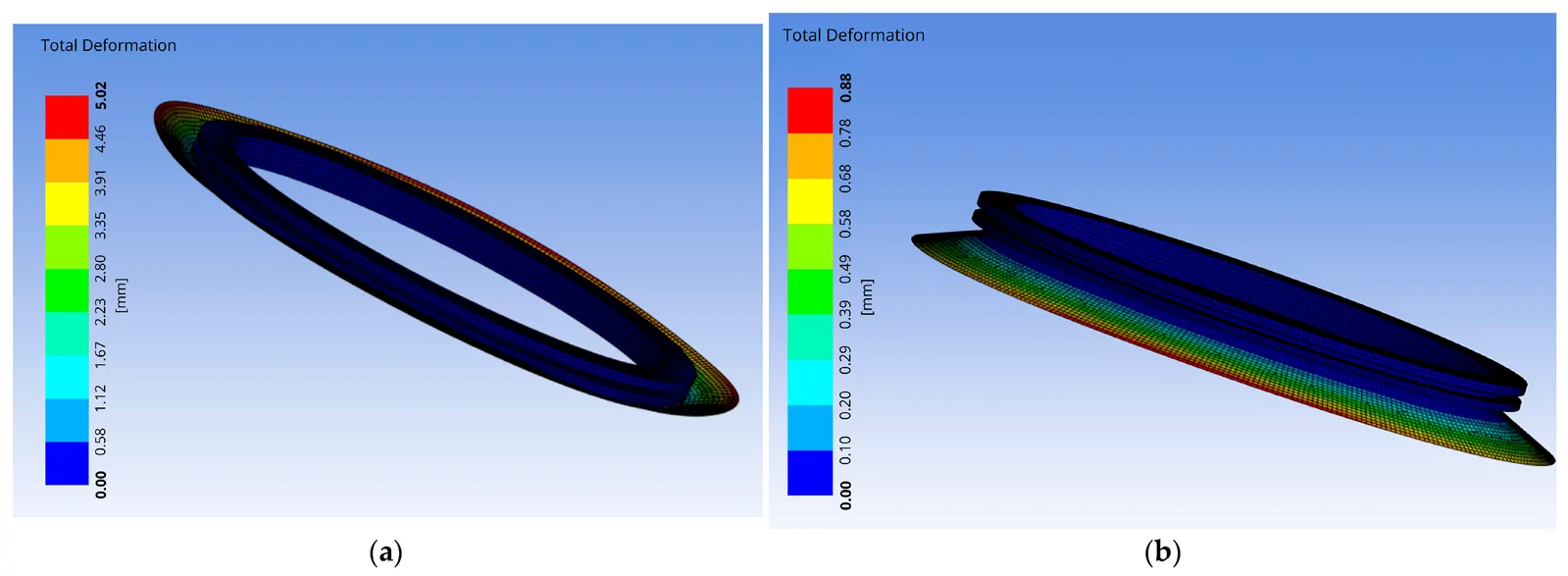
SEBS total deformation under constant pressure of 25,000 Pa: (**a**) Variant 1 Shore A 20; (**b**) Variant 1 Shore A 65.

Deformation of SEBS gaskets showed a clear exponential dependence on Shore A hardness (Figure 8). At the highest pressure of 25,000 Pa, SEBS Shore A 20 underwent excessively large deformation (e.g., compared to the stiffest material), confirming its unsuitability for this application (Figure 9). The results support the established optimal hardness range of Shore A 30–50 for balancing comfort and sealing performance in swimming goggles.

**Figure 10:**
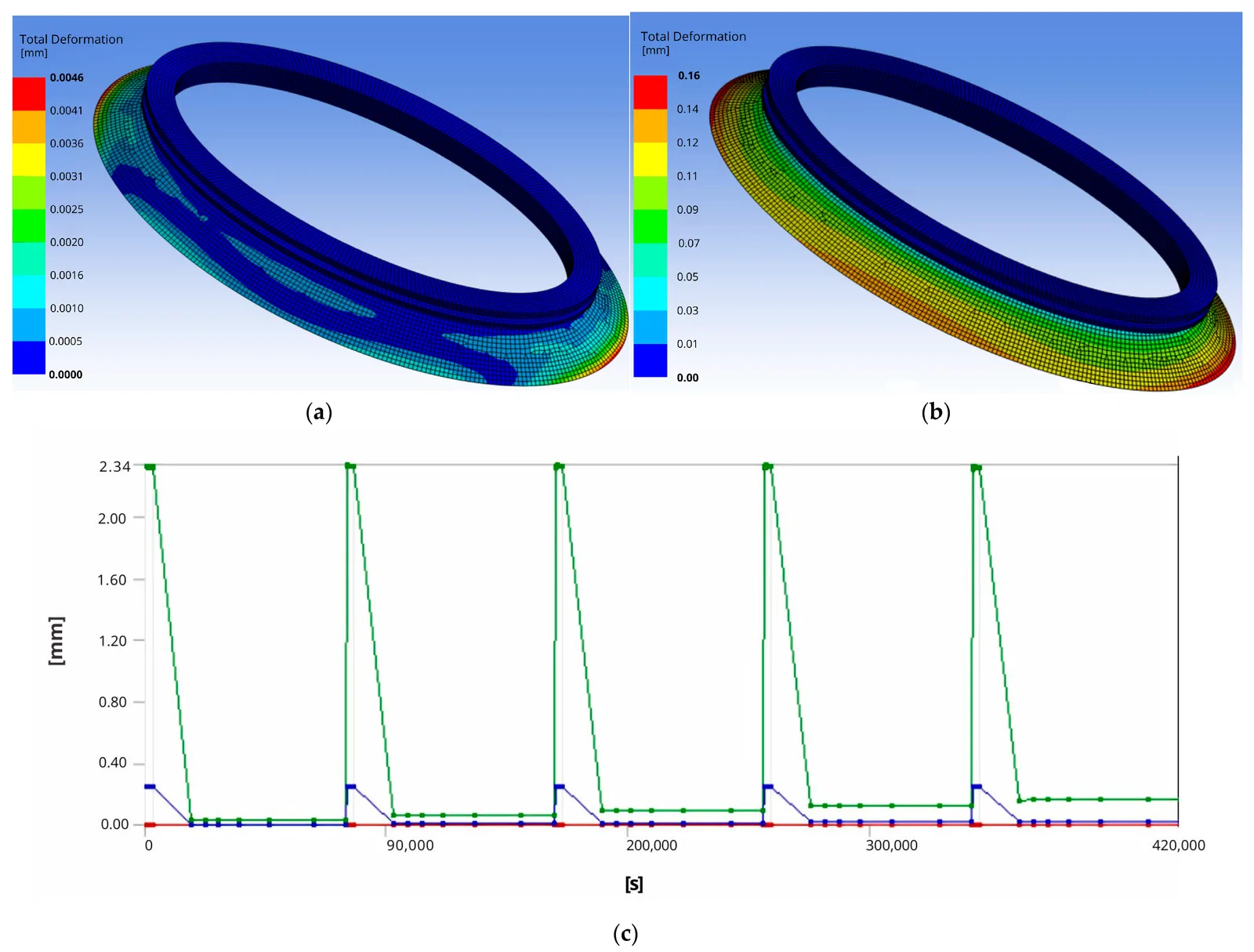
Cyclic relaxation test results: (**a**) total deformation of silicone Shore A 40 (end of the last relaxation cycle); (**b**) total deformation of SEBS Shore A 40 (end of the last relaxation cycle); (**c**) graph of changes in minimum (red color), maximum (green color), and average (blue color) deformation of SEBS Shore A 40 over time.

Cyclic relaxation tests (five cycles of 1 h at 25,000 Pa followed by 23 h recovery) revealed pronounced differences in long-term dimensional stability. After cycling, SEBS Shore A 40 exhibited a permanent set of 0.16 mm, whereas silicone showed only 0.0046 mm (Figure 10).

**Figure 11:**
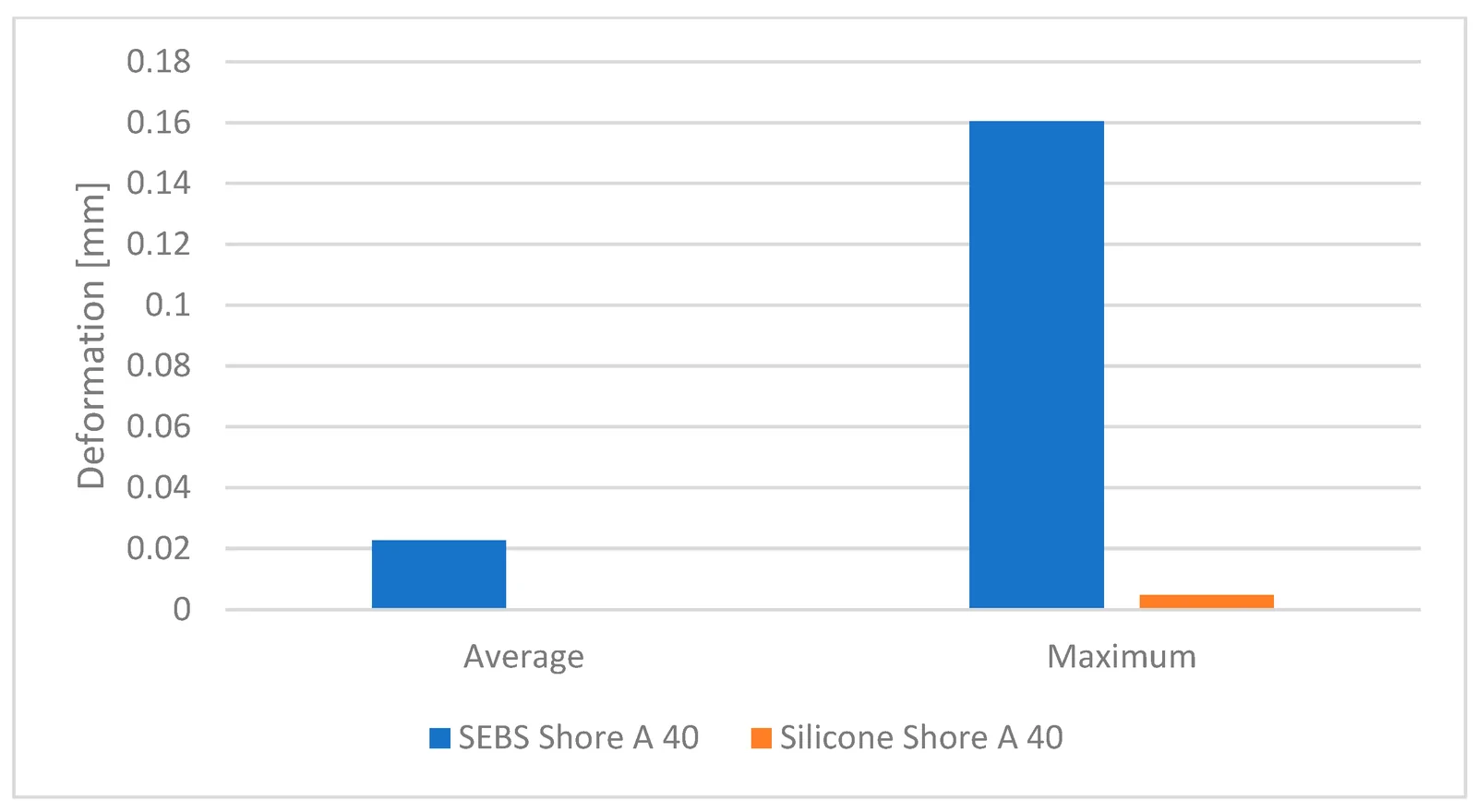
Graphical description of average and maximum deformation at the end of the last cycle of cyclic relaxation.

Although both values are small in absolute terms, the nearly 35-fold difference is significant (Figure 11). Silicone demonstrated markedly superior shape recovery, consistent with its inherently lower compression set and reduced stress relaxation compared with thermoplastic elastomers such as SEBS. This behavior stems from the stable siloxane backbone of silicone, which limits viscous flow and chain rearrangement under sustained compression.

Within the adopted five-cycle loading–recovery protocol, SEBS Shore A 40 exhibited greater residual deformation than silicone Shore A 40, indicating lower shape recovery under the modeled conditions. Because the geometry, mesh, boundary conditions, pressure level, and loading–recovery history were identical for both materials, the difference between their predicted responses primarily reflects the adopted viscoelastic parameter sets. In particular, the SEBS model combined a lower long-term shear-modulus ratio with substantially longer relaxation times and therefore retained a greater time-dependent response between consecutive cycles.

The absolute magnitude and spatial distribution of the residual deformation nevertheless remain dependent on the selected gasket geometry and loading protocol. Changes in pressure magnitude, loading duration, recovery time, or boundary conditions could alter the predicted response. Since no separate sensitivity analysis was conducted, the individual quantitative contributions of the Prony parameters, geometry, and loading conditions cannot be distinguished. The results should therefore be interpreted as a comparative, model-dependent assessment. A larger number of experimentally validated cycles would be required to determine whether the predicted difference translates into reduced sealing performance during long-term use.

### 4.2. Material Sensitivity Analysis

The material sensitivity analysis was performed for the SEBS model under the uniform pressure of 25 kPa, representing the more severe of the two uniform-pressure conditions. Three initial stiffness assumptions were considered: a soft Shore A20 variant, the experimentally informed reference variant, and a stiff Shore A40 variant.

Within the 25 kPa sensitivity simulations, the global response was compared at a common reaction force of F_z_ = 14.936 N. The corresponding displacements were 0.534 mm for the Shore A20 variant, 0.373 mm for the reference variant, and 0.225 mm for the Shore A40 variant. The predicted displacement therefore decreased monotonically with increasing initial shear modulus. The corresponding secant stiffness values were 27.97, 40.04, and 66.38 N/mm, respectively. The sensitivity of the global response was thus regular and mechanically consistent.

The spatial extent of large local compression was additionally evaluated using the minimum principal logarithmic strain. The logarithmic strain was converted to positive engineering compressive strain using the standard formulation:

(5) $$c_{e}=1-exp(\varepsilon_{3,e}),$$

where *ε*_3,*e*_ < 0 is the minimum principal logarithmic strain.

Because the mesh contained elements of different volumes, volume-weighted statistics were used as the primary measures. These compression-based quantities are distinct from the equivalent-strain values reported for the general loading analysis.

Under the 25 kPa condition, the volume-weighted mean compressive strain was 1.24% for the reference SEBS model, and only 0.13% of the SEBS volume exceeded 30% compression. Thus, approximately 99.87% of the material volume remained below this threshold. For the Shore A40 variant, the volume-weighted mean compressive strain was 0.81%, and no material volume exceeded 30% compression. In contrast, the Shore A20 variant exhibited a volume-weighted mean compressive strain of 11.83%, with 11.45% of the SEBS volume exceeding 30% compression.

The results indicate that the high local compressive strains in the reference model were confined to a very small portion of the gasket volume, even under the more severe uniform-pressure condition. However, the Shore A20 variant showed that the extent of large deformation increases substantially when a considerably softer material assumption is adopted. The sensitivity analysis therefore defines the response range associated with uncertainty in the assumed SEBS stiffness. It does not constitute a comparison or validation of different constitutive formulations.

### 4.3. Gasket Material Hardness

**Figure 12:**
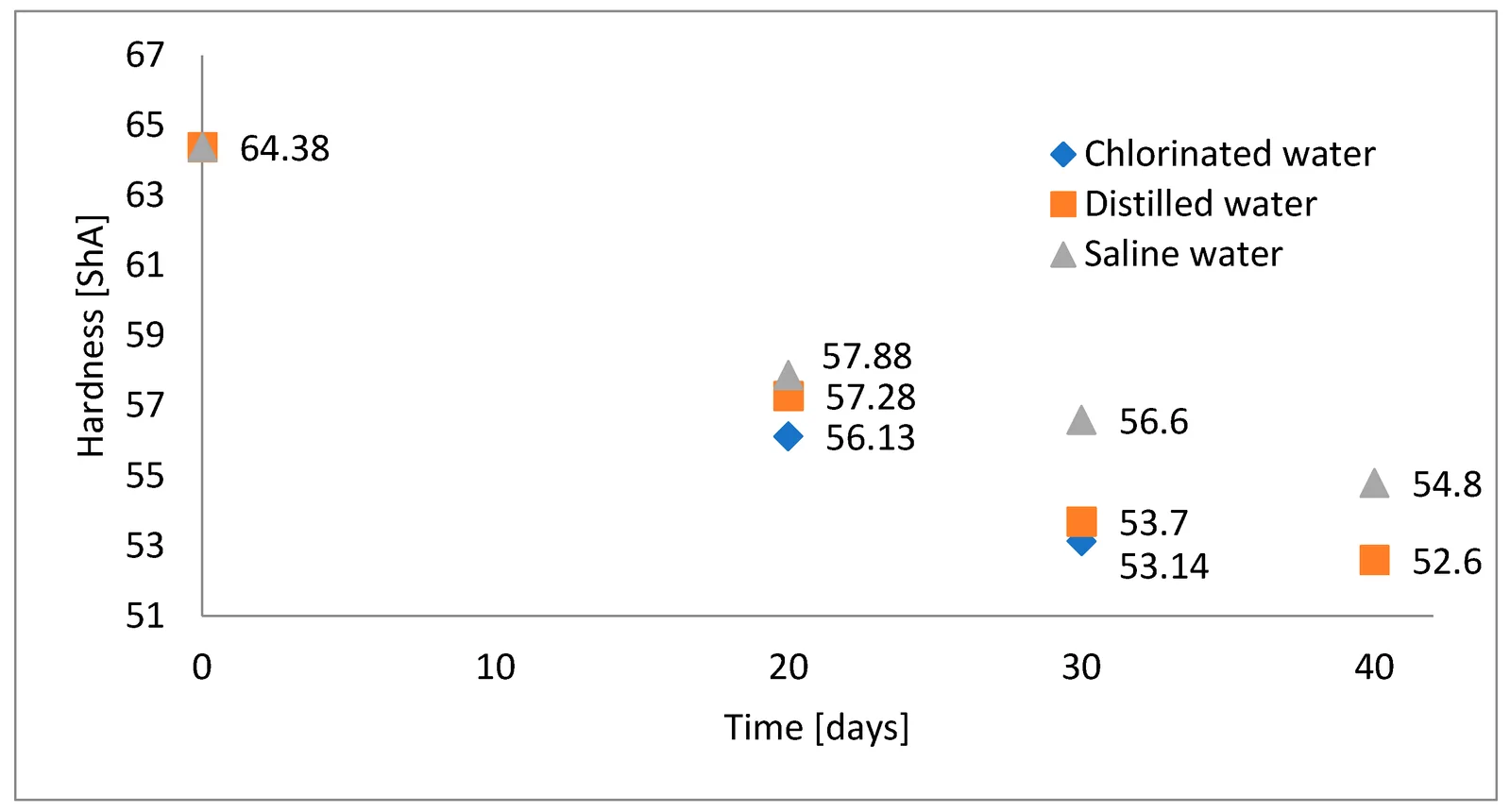
Gasket material hardness distribution vs. time.

Gasket material hardness was measured after various periods of water conditioning. The hardness distribution of the samples for different types of water as well as the statistical analysis are presented as a function of time in Figure 12 and Table 6, Table 7 and Table 8.

A clear, though varying, decrease in hardness is observed for all samples during conditioning. In the case of saltwater, the hardness decreases almost linearly; for the maximum conditioning time, it drops by 14.88% to 85.12% of the initial value.

The hardness profiles of samples conditioned in chlorinated and distilled water are similar to each other and strongly nonlinear. Between 30 and 40 days of conditioning, the hardness profile approaches an asymptotic behavior. For chlorinated water, the hardness drops by 17.46% (to 82.54%) after 30 days and by 18.30% (to 81.70%) after 40 days of conditioning. For distilled water, the corresponding decreases are 16.59% and 18.30%, respectively.

Since the measurements for chlorinated, distilled, and saline water were obtained from independent samples, one-way analysis of variance (one-way ANOVA) was performed separately for the measurements collected after 20, 30, and 40 days. In all three cases, the effect of water type on the measured parameter was statistically significant: 20 days, *p* < 0.0001; 30 days, *p* < 0.0001; 40 days, *p* < 0.0001. Post hoc analysis using Tukey’s HSD test revealed that after 20 days, statistically significant differences were observed between chlorinated and distilled water and between chlorinated and saline water, whereas the difference between distilled and saline water was not statistically significant. After 30 days, statistically significant differences were found for all pairwise comparisons, i.e., between chlorinated and distilled water, chlorinated and saline water, and distilled and saline water. After 40 days, no statistically significant difference was observed between chlorinated and distilled water, while the saline water group differed significantly from both the chlorinated and distilled water groups.

### 4.4. Tensile Strength of the Gasket Material

**Figure 13:**
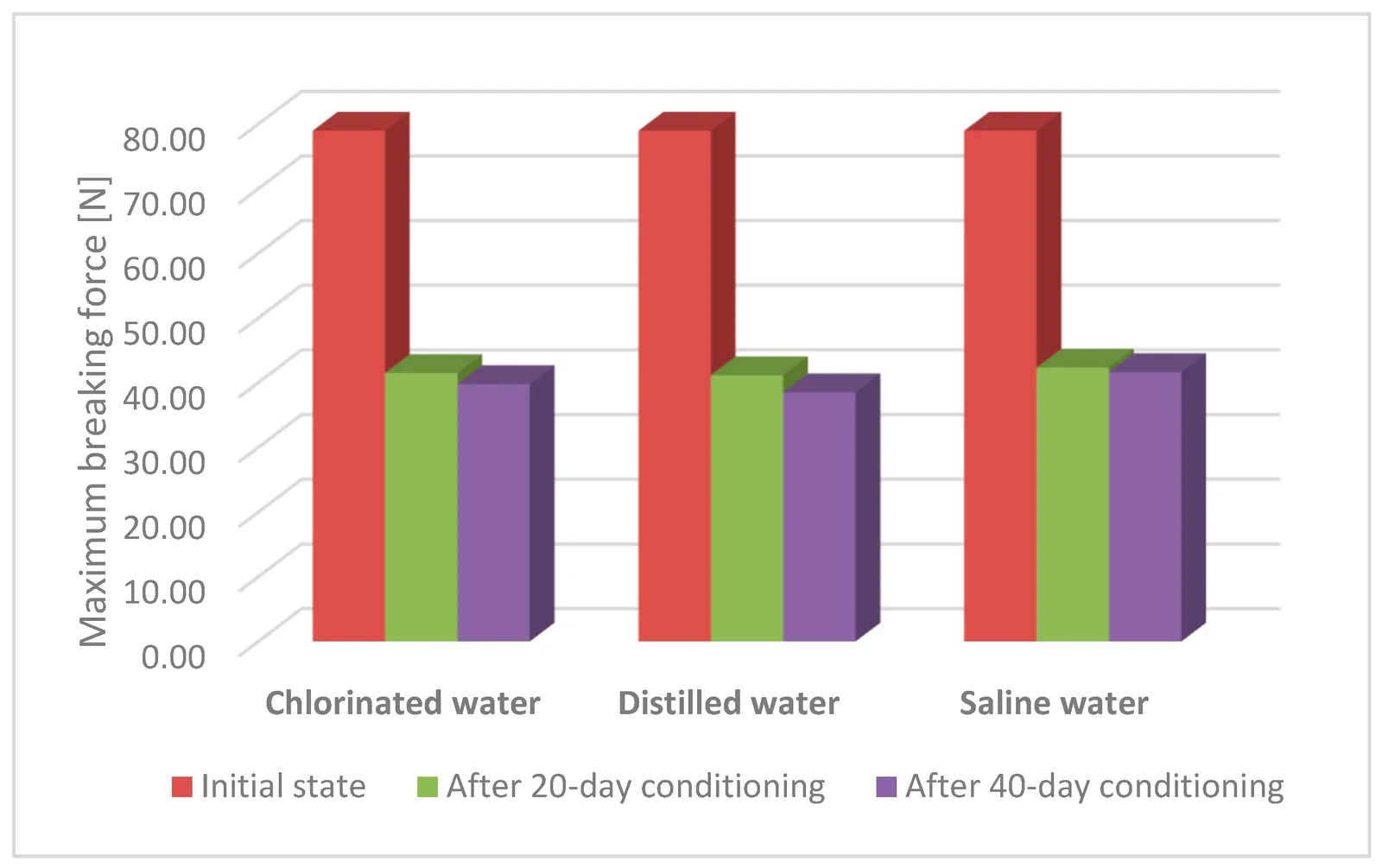
Maximum breaking force vs. conditioning time.

The maximum breaking force is illustrated in Figure 13, with corresponding relative values provided in Table 9.

The results indicate a clear decrease in force following conditioning. The greatest reduction occurs after 20 days, whereas values stabilize after 40 days. The values are comparable regardless of the conditioning environment.

The statistical analysis of obtained results is presented in Table 10, Table 11 and Table 12.

One-way ANOVA was performed separately for the measurements obtained after 20 and 40 days using independent samples. After 20 days, no significant effect of water type on the relative maximum breaking force was observed (*p* > 0.05). After 40 days, the effect of water type was statistically significant (*p* < 0.0001). Tukey’s HSD post hoc test demonstrated that after 40 days, all pairwise comparisons (chlorinated vs. distilled, chlorinated vs. saline, and distilled vs. saline) were statistically significant.

### 4.5. Relative Elongation at Break of the Gasket Material

**Figure 14:**
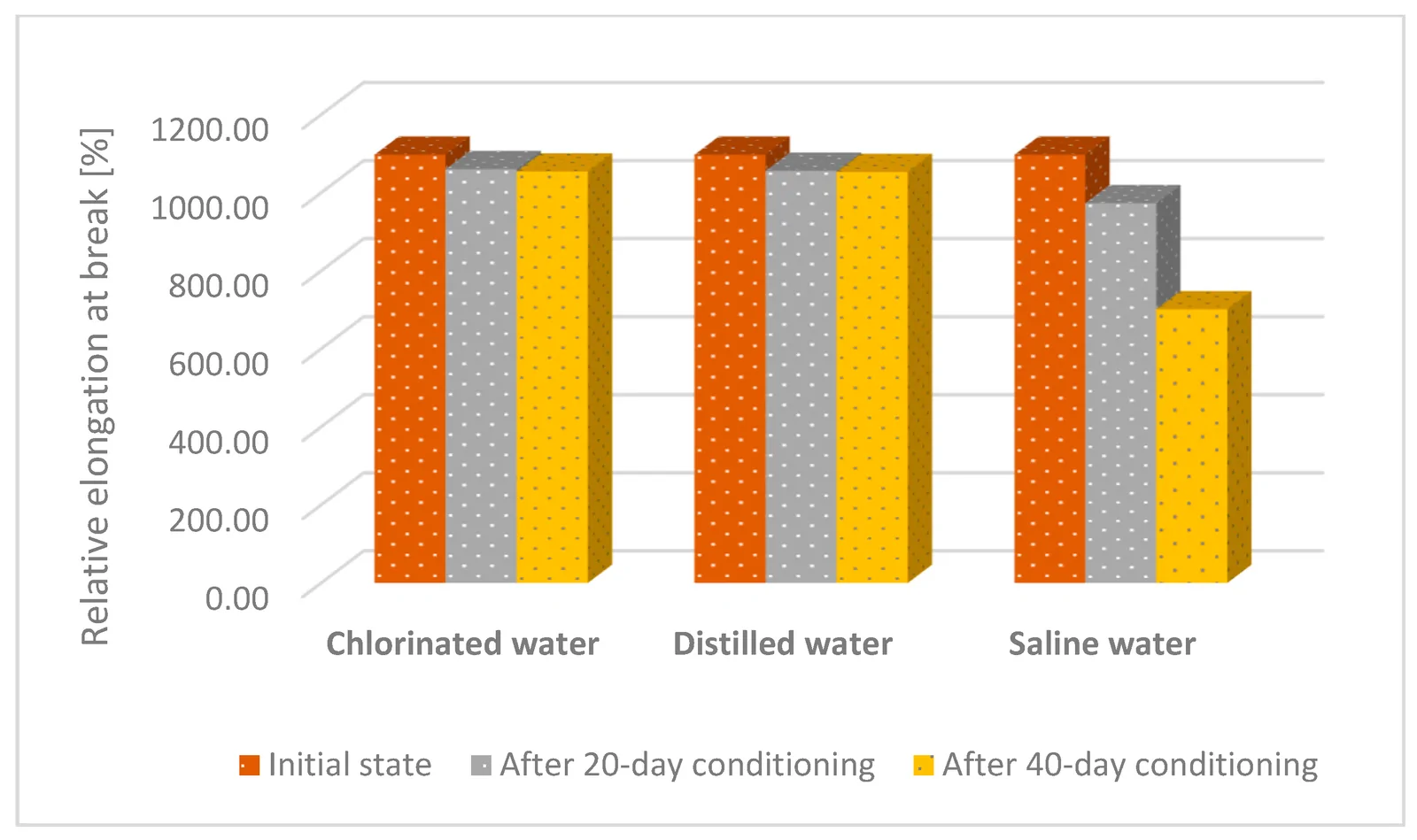
Maximum relative elongation at break vs. conditioning time.

**Figure 15:**
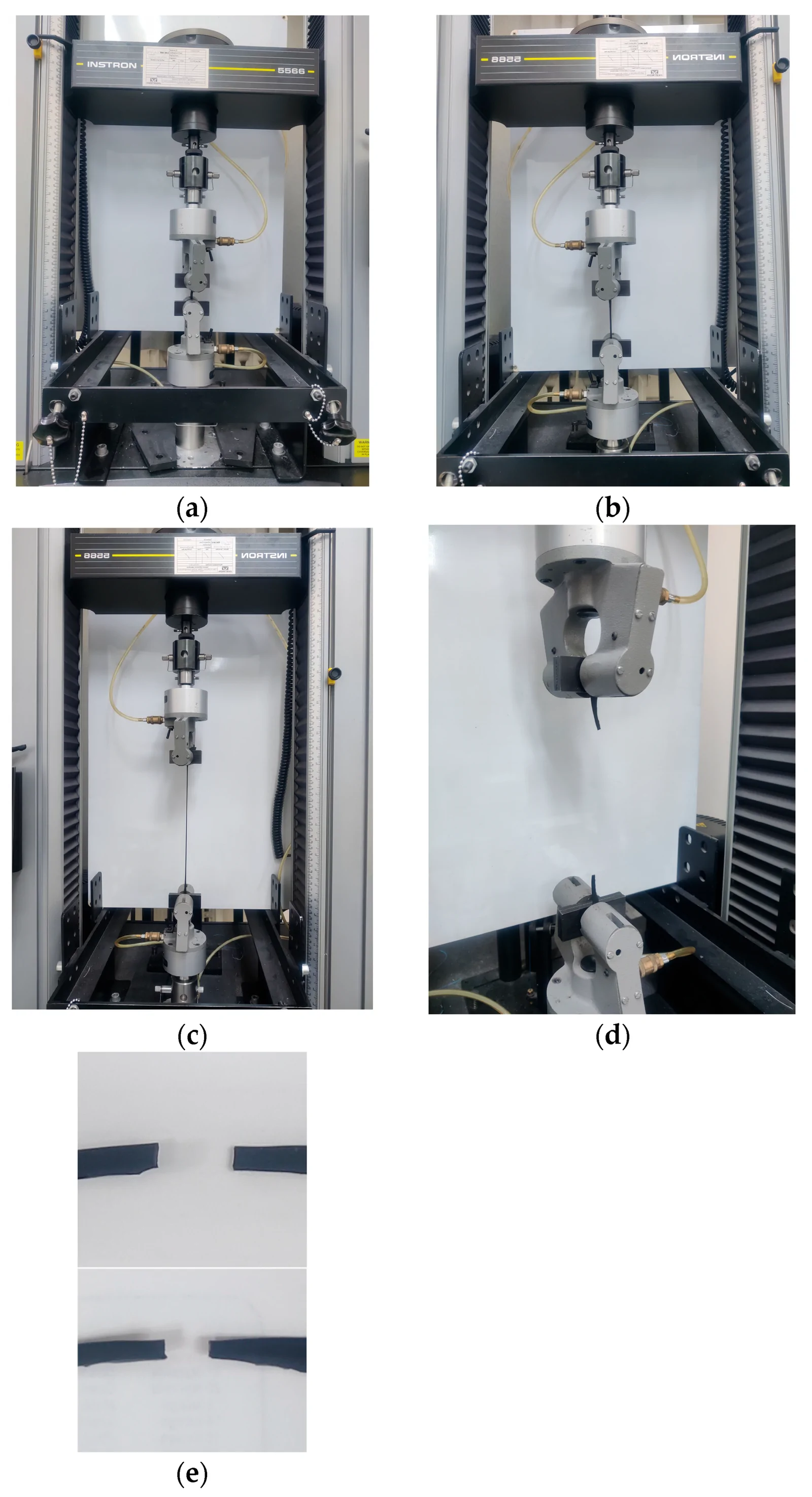
(**a**–**d**) Various stages of the gasket material specimen during tensile testing on an Instron universal testing machine; (**e**) representative fracture of the specimen.

The relative elongation at break is illustrated in Figure 14, with corresponding relative values provided in Table 13. The statistical analysis of obtained results is presented in Table 14, Table 15 and Table 16. The individual stages of material tensile deformation up to complete failure are presented in Figure 15a–d. Specimen rupture consistently occurs in a similar manner, approximately perpendicular to its longitudinal axis. The fracture of the gasket material specimen is illustrated in Figure 15e.

In the case of chlorinated and distilled water, the results are comparable before and after conditioning, with maximum decreases of approximately 4% after 40 days. In contrast, saline water conditioning leads to a distinct drop in elongation. Reductions in elongation at break for saline water reach 11.31% after 20 days and 36.11% after 40 days. These results demonstrate that saline water has a significant impact on the specimen’s elongation.

The study demonstrates that the conditioning environment significantly affects the material properties. Chlorinated and distilled water substantially reduce the breaking force without causing changes in the relative elongation at break. Saline water causes the most severe degradation of the material. The breaking force is comparable to the previous cases, but the specimen breaks at an elongation that is 36.11% lower after the maximum conditioning period.

The statistical analysis as a function of time is presented in Table 14, Table 15 and Table 16.

One-way ANOVA was performed separately for the measurements obtained after 20 and 40 days using independent samples. A statistically significant effect of water type on the relative elongation at break was observed after both 20 days (*p* < 0.0001) and 40 days (*p* < 0.0001). Tukey’s HSD post hoc test showed that the chlorinated and distilled water groups did not differ significantly at either time point, whereas the saline water group differed significantly from both chlorinated and distilled water after both 20 and 40 days.

## 5. Discussion and Conclusions

Numerical simulations revealed important differences between the investigated materials and geometries. Although SEBS Shore A 40 provided good initial sealing performance, cyclic loading simulations showed a substantially higher permanent set (0.16 mm) compared to silicone (0.0046 mm) after five loading–recovery cycles. This nearly 35-fold difference indicates that silicone exhibits markedly superior long-term dimensional stability, making it more suitable for durable swimming-goggle applications. Furthermore, the comparison of two gasket geometries clearly showed that differences in geometry can significantly affect conformal contact with the facial surface, despite similar absolute deformation values. This highlights that proper gasket geometry design is critical for achieving reliable sealing performance.

The results of hardness, tensile strength, and elongation-at-break tests demonstrate that prolonged exposure of swimming-goggle gaskets to different aqueous environments leads to measurable degradation of material properties. Conditioning in chlorinated, distilled, and saline water caused reduction in both Shore A hardness (by 11–18% after 40 days) and maximum breaking force (by approximately 47–51% after 40 days). The most pronounced effect on elongation at break was observed in saline water, with a decrease of up to 36% after 40 days of immersion. These findings confirm that the type of environment has a differentiated impact on the material degradation.

The experimental and numerical parts of the study should be interpreted as complementary rather than directly coupled. The conditioning experiments quantify changes in selected macroscopic properties of commercial SEBS gaskets, whereas the finite-element simulations evaluate the comparative influence of material stiffness and gasket geometry under prescribed nominal loading conditions.

The obtained results are consistent with previous studies on elastomer degradation in various environments. In this paper, the tensile strength in a uniaxial tensile test was adopted as the measure of gasket material loading. Other authors use different dynamic parameters to define the material. von Mises stress can be used because marine sealing systems can be simultaneously compressed, stretched, and sheared in multiple directions in a saltwater environment, as noted by Xinkai Ding et al. [1]. The results show that long-term aging significantly deteriorates sealing performance, with notable reductions in contact pressure and von Mises stress, ultimately affecting system reliability. According to Wang et al. [2], the parameter for O-rings is the compression ratio. The obtained trajectories of the compression ratio as a function of aging show an asymptotic decrease over time. Most authors account for the effects of temperature [3,4,5,6] or other factors (oil, oxidative aging) [13,14,15] on elastomer aging. However, this is irrelevant for swimming goggles, where the temperature is low and nearly constant. Regardless of the aging conditions applied, a decrease in the values chosen as representative was always observed.

The present work combined numerical analysis with experimental conditioning to evaluate material degradation and gasket behavior under representative loading scenarios. However, several limitations should be acknowledged. The conditioning was performed by static immersion at a constant room temperature, without simultaneous cyclic mechanical deformation, wet–dry cycles, controlled UV exposure, temperature fluctuations, exposure to sweat, sebum, or sunscreen, or temporal variations in chlorine concentration. This relatively mild conditioning protocol may also explain why the SEM observations performed after conditioning did not reveal sufficiently distinct and systematic surface changes to support a detailed microstructural analysis; therefore, these results were not included in the present study. Surface SEM imaging alone may not be sufficiently sensitive to detect chemical or molecular-level changes associated with polymer degradation, such as oxidation, chain scission, changes in phase morphology, or the loss or migration of material constituents. The impact of conditioning on the material microstructure therefore remains unresolved and requires systematic investigation using complementary techniques such as SEM combined with FTIR, thermal analysis, or other appropriate material-characterization methods. The numerical model used prescribed distributed pressure instead of explicit contact with compliant facial tissues. The material parameters were primarily adopted from the literature, while only the initial stiffness of the reference SEBS was approximately estimated from the available graphical uniaxial tensile response. Because the original numerical data were unavailable, a formal fit of the complete nonlinear response and a reliable quantitative fitting error could not be obtained. Consequently, the model does not directly predict the actual gasket–skin contact pressure or sealing performance, and the results should be interpreted primarily as a comparative assessment of the considered materials and geometries rather than as quantitatively calibrated predictions of gasket–skin interaction or sealing performance. The localized peak strains obtained under tight-fit and variable-pressure conditions may be more sensitive to the selected Neo-Hookean formulation than the average deformation response. As the first stage of a broader research program, the present study provides a basis for future work combining environmental conditioning with cyclic mechanical loading, systematic microstructural characterization, material-specific parameter identification, and closer experimental validation of the numerical model.

Future research should develop accelerated aging protocols that better reflect real swimming conditions, i.e., incorporate cyclic mechanical loading during conditioning and consider the combined effect of sweat and sebum. In addition, it would be valuable to extend the analysis towards the hydrodynamic and aerodynamic performance of the entire goggle system. Potential solutions to some of the problems proposed may build upon ideas developed in earlier publications [29,30,31,32].

Nevertheless, the obtained results provide useful insights into the relative performance of neoprene, silicone, and SEBS gaskets and underline the importance of material selection and geometry optimization in the design of long-lasting swimming goggles. This could facilitate accelerated material conditioning, enable the optimization of these materials based on various properties, and provide a framework for further research in this field.

## Figures and Tables

**Table 1:** Mechanical parameters of basic gasket materials.

Polymer	Tensile Strength, MPa	Young Modulus, MPa	Elongation at Break, %	Hardness, ShA	Density, g/cm^3^
SEBS, 100%	6.00–7.06	0.43	800–2382	30–50	0.94
Neoprene	27.579	6.136	600	35–95	1.23
Silicone	5–10	1–50	200–800	25–95	1.1–2.3

**Table 2:** Geometry variant descriptions.

Geometry Variant	Description	Upper Contour Shape	Bounding Box	Total Volume
1	Consists of three body groups, predominantly modeled as a thin-walled surface body incorporating both membrane and bending stiffness behaviors (Membrane and Bending).	B-type	X = 50 mm, Y = 30 mm, Z = 5.4 mm.	1.685 × 10^−6^ m^3^ (≈1.68 cm^3^).
2	Consists of a single solid body (Solid), modeled with full 3D continuum elements.	P-type	X = 50 mm, Y = 30 mm, Z = 4.1 mm.	1.348 × 10^−6^ m^3^ (≈1.35 cm^3^).

**Table 3:** Neo-Hookean material parameters.

Material	Initial Shear Modulus *μ*, MPa	Incompressibility Parameter D_1_, MPa^−1^
Neoprene Shore A 40	0.65	0.01
Silicone Shore A 40	0.66	0.01
SEBS Shore A 20	0.28	0.01
SEBS Shore A 40	0.66	0.01
SEBS Shore A 50	1.00	0.006
SEBS Shore A 65	1.80	0.003

**Table 4:** Estimation of contact pressure from measured strap elongation (SEBS Shore A 40 strap, *E* = 1.98 MPa) *.

Fit Condition	*L*_0_ [cm]	*L* [cm]	Δ*L* [cm]	Engineering Strain *ε*	Tensile Force *F* [N]	Contact Pressure *P* [kPa]
Optimal (comfortable and secure seal)	38.0	40.0	2.0	0.05	3.65	≈5
Tight (excessive compression and discomfort)	31.0	40.0	9.0	0.29	20.12	≈25
High localized transient stress concentrations during the top-down gasket donning process	n/a	n/a	n/a	n/a	n/a	Variable: 0 kPa to 40 kPa

* Parameters used: (a) effective Young’s modulus *E* = 1.98 MPa (consistent with *μ* = 0.66 MPa from Table 3 via *E* ≈ 3*μ*); (b) strap cross-section A = 35 mm^2^; (c) gasket–face contact area (one side) ≈ 8 cm^2^.

**Table 5:** Numerical results of average and maximum values of deformation, stress, and strain.

Geometry Variant	Material Shore A 40	Pressure [Pa]	Deformation [mm]		Stress [MPa]		Strain [%]	
			Av.	Max.	Av.	Max.	Av.	Max.
Variant 1	Neoprene rubber	5000	0.04	0.48	0.01	0.11	0.74	5.43
		25,000	0.26	2.37	0.07	0.64	3.77	30.96
		Variable	0.09	3.60	0.03	0.87	1.53	40.75
	Silicone	5000	0.05	0.47	0.01	0.11	0.73	5.34
		25,000	0.25	2.34	0.07	0.64	3.71	30.45
		Variable	0.09	3.56	0.03	0.89	1.51	40.25
	SEBS	5000	0.05	0.47	0.01	0.11	0.73	5.34
		25,000	0.25	2.34	0.07	0.64	3.71	30.45
		Variable	0.09	3.56	0.03	0.89	1.51	40.25
Variant 2	SEBS	5000	0.04	0.43	0.01	0.10	0.63	5.28
		25,000	0.22	2.22	0.06	0.49	3.33	25.02

**Table 6:** Statistical analysis of material hardness measurements—chlorinated water.

Statistical Analysis	Conditioning Time [Days]		
	20	30	40
Mean value	56.13	53.14	52.60
Variation	1.011	1.020	1.245

**Table 7:** Statistical analysis of material hardness measurements—distilled water.

Statistical Analysis	Conditioning Time [Days]		
	20	30	40
Mean value	57.28	53.70	52.60
Variation	1.015	1.025	1.347

**Table 8:** Statistical analysis of material hardness measurements—saline water.

Statistical Analysis	Conditioning Time [Days]		
	20	30	40
Mean value	57.88	56.60	54.80
Variation	1.129	1.245	1.501

**Table 9:** Relative maximum breaking force after specified conditioning time compared to initial values.

Water Type	Relative Maximum Breaking Force After Conditioning Time [%]	
	20 Days	40 Days
Chlorinated	52.53	50.38
Distilled	52.10	48.78
Saline	54.86	52.72

**Table 10:** Statistical analysis of breaking force measurements—chlorinated water.

Statistical Analysis	Conditioning Time [Days]		
	0	20	40
Mean value	78.96	41.48	39.78
Variation	8.211	3.936	3.881

**Table 11:** Statistical analysis of breaking force measurements—distilled water.

Statistical Analysis	Conditioning Time [Days]		
	0	20	40
Mean value	78.96	41.14	38.52
Variation	8.211	4.138	1.995

**Table 12:** Statistical analysis of breaking force measurements—saline water.

Statistical Analysis	Conditioning Time [Days]		
	0	20	40
Mean value	78.96	42.32	41.63
Variation	8.211	4.001	2.045

**Table 13:** Relative maximum relative elongation at break after specified conditioning time compared to initial values.

Water Type	Relative Elongation at Break After Conditioning Time [%]	
	20 Days	40 Days
Chlorinated	96.61	96.05
Distilled	96.24	95.98
Saline	88.69	63.89

**Table 14:** Statistical analysis of relative elongation at break measurements—chlorinated water.

Statistical Analysis	Conditioning Time [Days]		
	0	20	40
Mean value	1097.21	1060.00	1053.83
Variation	155.722	162.009	195.113

**Table 15:** Statistical analysis of relative elongation at break measurements—distilled water.

Statistical Analysis	Conditioning Time [Days]		
	0	20	40
Mean value	1097.21	1055.97	1053.07
Variation	155.720	201.112	172.981

**Table 16:** Statistical analysis of relative elongation at break measurements—saline water.

Statistical Analysis	Conditioning Time [Days]		
	0	20	40
Mean value	1097.21	973.15	701.01
Variation	155.724	121.012	79.09

## Data Availability

The raw data supporting the conclusions of this article will be made available by the authors on request.
